# Determinants of non- response to a second assessment of lifestyle factors and body weight in the EPIC-PANACEA study

**DOI:** 10.1186/1471-2288-12-148

**Published:** 2012-09-24

**Authors:** Anne M May, Lotte E Adema, Dora Romaguera, Anne-Claire Vergnaud, Antonio Agudo, Ulf Ekelund, Annika Steffen, Philippos Orfanos, Nadia Slimani, Sabina Rinaldi, Traci Mouw, Sabine Rohrmann, Silke Hermann, Heiner Boeing, Manuela M Bergmann, Marianne Uhre Jakobsen, Kim Overvad, Nicholas J Wareham, Carlos Gonzalez, Anne Tjonneland, Jytte Halkjaer, Timothy J Key, Elizabeth A Spencer, Veronica Hellstrom, Jonas Manjer, Bo Hedblad, Eiliv Lund, Tonje Braaten, Françoise Clavel-Chapelon, Marie-Christine Boutron-Ruault, Laudina Rodríguez, Maria J Sánchez, Miren Dorronsoro, Aurelio Barricarte, Jose Maria Huerta, Androniki Naska, Antonia Trichopoulou, Domenico Palli, Valeria Pala, Teresa Norat, Amalia Mattiello, Rosario Tumino, Daphne van der A, H Bas Bueno-de-Mesquita, Elio Riboli, Petra HM Peeters

**Affiliations:** 1 Julius Centre for Health Sciences and Primary Care, University Medical Centre Utrecht, Utrecht University, PO Box 85500, 3508, GA, Utrecht, The Netherlands; 2 National Institute for Public Health and the Environment (RIVM), Bilthoven, The Netherlands; 3 Department of Epidemiology and Biostatistics, School of Public Health, Imperial College London, London, United Kingdom; 4 Unit of Nutrition, Environment and Cancer, Catalan Institute of Oncology, IDIBELL, Barcelona, Spain; 5 Medical Research Council, Epidemiology Unit, Cambridge, United Kingdom; 6 German Institute of Human Nutrition Potsdam-Rehbrücke, Heidelberg, Germany; 7 Hellenic Health Foundation, Athens, Greece AND Department of Hygiene, Epidemiology and Medical Statistics, School of Medicine, University of Athens, Athens, Greece; 8 International Agency for Research on Cancer (IARC-WHO), Lyon, France; 9 Division of Clinical Epidemiology, German Cancer Research Center, Heidelberg, Germany; 10 Department of Epidemiology, School of Public Health, Aarhus University, Aarhus, Denmark; 11 Department of Cardiology, Aalborg Hospital, Aarhus University Hospital, Aalborg, Denmark; 12 Danish Cancer Society, Institute of Cancer Epidemiology, Copenhagen, Denmark; 13 Cancer Research UK Epidemiology Unit, University of Oxford, Oxford, United Kingdom; 14 Department of Public Health and Clinical Medicine, Nutritional Research, Umeå University, Umeå, Sweden; 15 Department of Surgery, Malmø University Hospital, Lund University, Malmø, Sweden; 16 Department of Clinical Science, Malmø University Hospital, Lund University, Malmø, Sweden; 17 Institute of Community Medicine, University of Tromsø, Tromsø, Norway; 18 Institut National de la Santé et de la Recherche Médicale (INSERM), ERI 20, EA 4045, Villejuif, France; 19 Institut Gustave Roussy, Villejuif, France; 20 Public Health and Participation Directorate, Health and Health Care Services Council, Asturias, Spain; 21 Andalusian School of Public Health, Granada, and CIBER de Epidemiology and Public Health (CIBERESP), Granada, Spain; 22 Public Health Department of Gipuzkoa, San Sebastian, Spain; 23 Public Health Institute of Navarra, Pamplona, Spain; 24 Epidemiology Department, Council of Health and Consumer Affairs, Murcia& CIBER Epidemiología y Salud Pública (CIBERESP), Murcia, Spain; 25 Molecular and Nutritional Epidemiology Unit, ISPO-Cancer Research and Prevention Institute, Florence, Italy; 26 Nutritional Epidemiology Unit, IRCCS Foundation, National Cancer Institute, Milan, Italy; 27 Dipartimento di Medicina Clinica e Sperimentale, Università di Napoli, Naples, Italy; 28 Cancer Registry, Azienda Ospedaliera "Civile M.P.Arezzo", Ragusa, Italy

**Keywords:** Non-response, Non-participation, Lost-to-follow-up

## Abstract

**Background:**

This paper discusses whether baseline demographic, socio-economic, health variables, length of follow-up and method of contacting the participants predict non-response to the invitation for a second assessment of lifestyle factors and body weight in the European multi-center EPIC-PANACEA study.

**Methods:**

Over 500.000 participants from several centers in ten European countries recruited between 1992 and 2000 were contacted 2–11 years later to update data on lifestyle and body weight. Length of follow-up as well as the method of approaching differed between the collaborating study centers. Non-responders were compared with responders using multivariate logistic regression analyses.

**Results:**

Overall response for the second assessment was high (81.6%). Compared to postal surveys, centers where the participants completed the questionnaire by phone attained a higher response. Response was also high in centers with a short follow-up period. Non-response was higher in participants who were male (odds ratio 1.09 (confidence interval 1.07; 1.11), aged under 40 years (1.96 (1.90; 2.02), living alone (1.40 (1.37; 1.43), less educated (1.35 (1.12; 1.19), of poorer health (1.33 (1.27; 1.39), reporting an unhealthy lifestyle and who had either a low (<18.5 kg/m2, 1.16 (1.09; 1.23)) or a high BMI (>25, 1.08 (1.06; 1.10); especially ≥30 kg/m2, 1.26 (1.23; 1.29)).

**Conclusions:**

Cohort studies may enhance cohort maintenance by paying particular attention to the subgroups that are most unlikely to respond and by an active recruitment strategy using telephone interviews.

## Background

Large cohort studies are subject to the problem of attrition. The most prominent types of attrition include those participants who have died during the follow-up period, those who cannot be located because of (e)migration, and those who do not respond to the follow-up survey (i.e., non-responders) [1,2]. Although some causes cannot be influenced by the researcher, study design and efforts to contact the study population can modify the degree of attrition [2].

High rates of non-participation to a follow-up survey can lead to selection bias, when the persons who drop-out differ significantly from the participants in characteristics that are related to the outcome being studied [3,4]. This loss of a selective group can reduce the external validity as well as the generalizability of the research findings [1,2,5,6]. The success of any longitudinal study, therefore, depends upon its participants remaining in the study [2]. Assessment of information on initial participation and retention rates helps to evaluate potential selection bias when non-participation during follow-up is not random [1,7,8]. Furthermore, assessment of determinants of attrition may identify characteristics of participants who are most unlikely to respond to the follow-up survey [1]. This may aid in management strategies to target specifically individuals with such characteristics and thus leading to reducing non-response [1,4,6,9]. Various population-based longitudinal cohort studies have shown that non-responders often differ from those who respond to a follow-up survey with respect to demographic, socioeconomic and health characteristics. Many factors have been investigated, though not all factors are consistently found to be significantly associated with non-response [10,11]. However, in most studies, non-responders are more likely to be among the youngest [1-3,12] or oldest participants [6,8,9], to live alone [1-4,6,9,13], to be less educated [1,4,6,8,11-14], unemployed [2,5,9,14] and to have a low income [5,6,11]. Non-responders are more likely to have an unhealthy lifestyle, especially being a smoker [2-4,7,8,11,13]. The general health profile of non-responders tends to be worse than that of responders [1,4,8,9,11,13,15,16] and a higher prevalence of obesity is observed [3,8,12].

To date, studies on determinants of non-response have been mainly conducted in single population-based cohorts where all participants were followed for the same time period. The present study, however, is based on data from almost 500.000 participants from 10 European countries, as part of the EPIC-PANACEA (**E**uropean **P**rospective **I**nvestigation into **C**ancer and Nutrition-**P**hysical **A**ctivity, **N**utrition, **A**lcohol, **C**essation of Smoking, **E**ating out of home **A**nd obesity) study. EPIC-PANACEA aims to investigate the determinants of obesity and body weight changes in Europe. For the purpose of EPIC-PANACEA data from a second assessment of body weight collected several years after baseline were centralized and combined with the EPIC baseline dataset. The length of follow-up as well as the method of contacting participants (i.e. by postal surveys, directly by phone or by a request to visit a study center for physical examination) differed between the collaborating centers. This allows insight in whether non-response differs with various methods of contacting participants and diverse durations of follow-up.

The purpose of the present study was twofold. First, we investigated whether baseline demographic, socio-economic, health variables, length of follow-up and method of contacting the participants predicted non-response to an invitation for a second assessment of lifestyle factors and body weight excluding those who were not (yet) contacted, and those who either died or emigrated during follow-up. This provides insight in important determinants of non-response that can be used to enhance cohort maintenance in future studies. Second, we compared all baseline participants for whom a second body weight assessment was missing (including non-responders, (e)migrated, deceased or not yet contacted participants) with responders, to evaluate whether the population lost to follow-up formed a selective group causing potential selection bias in future analyses.

## Methods

### Study population at baseline

The PANACEA study is part of the large EPIC study. EPIC is an ongoing multi-center prospective cohort study, designed to investigate the relationship of nutrition and lifestyle with cancer and other chronic diseases [17]. The study is conducted in several centers in ten European countries (Denmark [Copenhagen, Aarhus], France, Germany [Potsdam, Heidelberg], Greece, Italy [Florence, Varese, Ragusa, Turin, Naples], The Netherlands [Utrecht, Doetinchem, Amsterdam/Maastricht], Norway, Spain [Asturias, Granada, Murcia, Navarra, San Sebastian], Sweden [Malmø, Umea] and the United Kingdom [Oxford general health, Oxford health conscious, Cambridge]). In the present study, multiple centers within a country were treated as a single study center, when length of follow-up and data collection methods did not differ and when coordination took place out of one center. Therefore, data from multiple centers in Spain and Denmark are treated as single centers, whereas the centers from the UK, Germany, The Netherlands, Italy and Sweden are treated separately in our analyses. In Norway and Greece one coordinating center was situated.

Enrolment took place between 1992–2000, which resulted in recruitment of 521.448 male and female participants aged between 20–80 years. In many centers participants were invited from the general population residing in a given town or geographical area except for France where members of the health insurance for teachers were included; a large part of the Spanish and Italian centers included blood donors; the cohorts in Utrecht and Florence included women attending the breast cancer screening program. Half of the participants recruited by the Oxford centre are ‘health conscious’ vegetarian or healthy eaters partly recruited by contacting members of The Vegetarian Society of the UK and all surviving participants in the Oxford Vegetarian Study [18]. In France, Norway, Utrecht (The Netherlands) and Naples (Italy) only women were recruited. Participants were either invited by mail (Navarra and Asturias (Spain), Ragusa (Italy), France, Germany, Netherlands, United Kingdom, Denmark, Sweden, Norway), in person (Turin (Italy) or in person and by mail (Granada, Murcia and San Sebastian (Spain), Greece, Florence, Naples and Varese (Italy). Individuals who provided written informed consent were mailed standardized questionnaires on diet and on lifestyle, socio-economic and health variables. Most participants completed these questionnaires at home and were then invited to a study centre for an examination. In Spain and Ragusa (Italy), the participants received the non-dietary questionnaire by mail. The lifestyle questionnaire was self-administered when visiting the study centre, where also an interviewer-administered computer-driven dietary questionnaire was completed. Participants in Greece who were recruited in person completed an interviewer-administered questionnaire on diet and a questionnaire on lifestyle at the study centre. In Denmark and Malmö (Sweden), the participants filled in dietary questionnaires at home and lifestyle questionnaires at the study centres.

In all EPIC centres, at the study centre anthropometric measurements were performed and blood samples were taken. Only in France, Oxford-Health conscious group and Norway anthropometric measurements were self-reported by the participants. A detailed description of the data collection in each EPIC centre has been reported earlier [17].

Approval was obtained from the ethical review boards of the International Agency for Research on Cancer and from all local centers.

The EPIC-PANACEA project is designed to investigate the determinants of obesity and weight changes in Europe. From the 521.448 participants recruited initially, 23.479 participants were excluded because of missing information on dietary or lifestyle variables, unavailable information on body mass index (BMI), extreme values on anthropometry data, pregnancy or due to an extreme ratio between energy intake and energy requirement. Thus, 497.969 participants with complete baseline data on anthropometry were available for the baseline EPIC-PANACEA analyses.

### Follow-up data collection

EPIC participants are followed for vital status, cause of death and disease occurrence. In most of the centers these data are obtained by regular record linkage with the exception of Greece and Potsdam (Germany) where an active follow-up is used. For updating lifestyle and anthropometric data participants were contacted a second time several years after recruitment. For the purpose of EPIC-PANACEA the second assessment of body weight was centralized and combined with the baseline dataset.

Follow-up time between first and second anthropometry assessment differed between study centers due to logistical and financial reasons, and varied between two (in Heidelberg, Germany) to eleven (in Varese, Italy) years. Assessment was conducted through mailed questionnaires, with several exceptions: Spain and Greece contacted their participants by phone and also completed the questionnaire on the phone. Varese used a combination of postal survey and telephone interview. Cambridge (United Kingdom) and Doetinchem (i.e. a sub-cohort of the EPIC Bilthoven cohort, The Netherlands) invited their participants to come to the research center for a second measurement of anthropometry and other lifestyle factors.

In Ragusa (Italy), Turin (Italy) and Potsdam follow-up assessment is currently ongoing. Data from Ragusa and Turin are therefore not included in the present study. Potsdam provided available data from participants who were contacted so far. Naples (Italy) took a random sample of 700 participants of their baseline population for the second assessment round. In Doetinchem 1,101 baseline participants were not contacted for the follow-up survey. Hence, from all baseline participants 25,355 participants had not (yet) been contacted for a second assessment.

### Missing data on follow-up assessment of body weight

**Figure 1:**
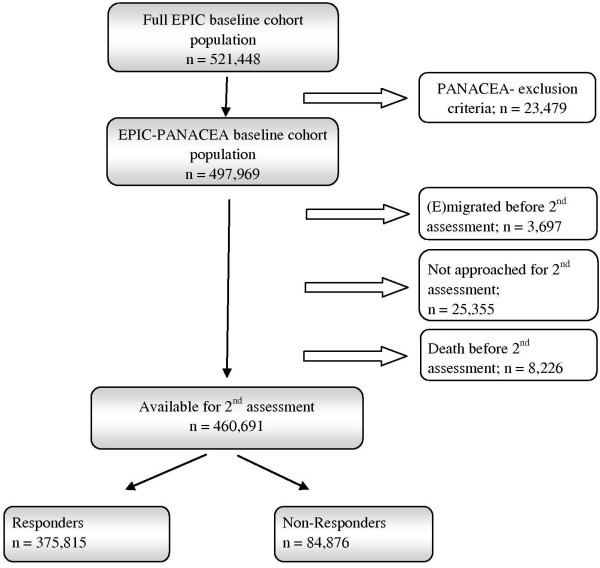
**Flow of participants through the EPIC-PANACEA study**.

In addition to the above mentioned reason for missing follow-up lifestyle and anthropometric data, i.e. not (yet) contacted, we defined three other reasons for missing data at follow-up, i.e. death (n = 8,226), (e)migration (this information was not provided by France, Spain, Greece and Germany) (n = 3,9697), and non-response to the invitation to participate in the second assessment round (n = 84,876). We defined non-responders as baseline participants who were contacted for the second assessment (alive and not (e)migrated), but who did not respond to the invitation to participate, i.e. a second assessment of body weight is not available in the EPIC-PANACEA dataset. We defined ‘persons with missing second assessment data’ as all persons for whom a second body weight assessment is missing (i.e. non responders, death, emigrated, or not contacted yet). Figure 1 summarizes the flow of the participants through the EPIC-PANACEA study.

### Potential determinants of non-response

Determinants were chosen based on their importance in future weight change analyses in EPIC-PANACEA or because they were related to non-response in other studies [1-9,12,13]. Selected demographic and socioeconomic variables included sex (male/female), age (≤40/41-60/>60 years), marital status (married or living together/single, divorced or separated, widowed) and highest educational level (primary school or less/vocational secondary school/other secondary school/college or university). Lifestyle variables included physical activity according to a validated physical activity index based on work and leisure time activity (inactive/moderately inactive/moderately active/active) [19], smoking status (never smoker/former smoker/current smoker) and alcohol consumption (non users/light alcohol users (0–18 g/day)/moderate alcohol users (18–60 g/day)/heavy alcohol users (>60 g/day)). Health variables included BMI (<18.5/18.5-25/25-30/≥30 kg/m^2^), having cancer or a chronic condition or disease (hypertension, stroke, myocardial infarction, diabetes mellitus) at baseline, or having developed cancer between baseline and the invitation for the second assessment of body weight. Finally recruitment strategy for the second assessment (postal survey, survey completed by telephone, request to visit a study center) and mean follow-up time (0–4 years, 4.1-8 years, >8 years) were taken into account.

### Data analysis

Response rates for the assessment of a second body weight were calculated according to the standard definitions used by the American Association for Public Opinion Research (AAPOR) [20]; i.e. the number of participants with a second weight assessment (responders) divided by the sum of the responders and participants who did not respond (non-responders), died, emigrated or were not yet contacted. Response rates were also calculated for the number of responders divided by the number of non-responders (i.e. excluding baseline participants who were not yet approached, died or emigrated before the second weight assessment from the denominator).

Baseline characteristics of responders (n = 375,815) were compared to characteristics of non-responders (n = 84,876). This information is important because it may help in defining strategies to prevent non-response. To predict probability of non-response by baseline characteristics a multivariate logistic regression model was used with response status (0 for response and 1 for non-response) as the dependent variable and all above mentioned baseline characteristics, recruitment strategy and mean follow-up time as independent variables. Odds ratios mutually adjusted for all variables and their 95% confidence intervals were calculated.

Secondly, baseline characteristics of responders (n = 375,815) were compared to characteristics of all those persons who had missing data for the second body weight assessment either because of death, (e)migration, not (yet) contacted or non-response (n = 122,154). This information is important in future analyses to interpret whether differences between cross-sectional relations in the baseline cohort (for example between physical activity and BMI) and results from longitudinal studies (for example relations between baseline physical activity and future weight change) can be explained by selection bias. Again a multivariate logistic regression model was used with response status as the dependent variable and above mentioned baseline characteristics as independent variables.

All analyses were performed with SPSS software, version 15.0 for Windows.

## Results

### Response

From all baseline participants (n = 497,969), a second assessment of weight was obtained for 375,815 persons (75.5%). When taking in the denominator only those persons who were contacted (n = 460,691 excluding deceased, (e)migrated and not approached persons) the response rate was 81.6%.

Table 1 shows the EPIC-PANACEA centers ranked according the time between first and second body weight assessment. Furthermore, the distribution of centers according to the different groups of attrition (i.e. not (yet) approached, deceased, (e)migrated, second assessment missing, non-response) is shown.

**Table 1:** Characteristics of the EPIC-PANACEA Cohort

**EPIC-PANACEA centers**^**a**^	**Total baseline population n**	**Mean age yrs (SD)**	**Mean follow-up time yrs (SD)**	**Method of contacting for 2**^**nd **^**assessment**	**Not (yet) contacted n (%)**	**Death n (%)**	**(E) migrated n (%)**	**2**^**nd**^** assessment missing**^**b**^**n (%)**	**Nonresponsec n (%)**
Heidelberg (GE)	24,954	51.0 (8.1)	2.1 (0.6)	P	0	129 (0.5)	na	1,771 (7.1)	1,642 (6.6)
Spain	40,418	49.3 (8.0)	3.3 (0.4)	T	0	202 (0.5)	na	703 (1.7)	501 (1.2)
France	71,910	52.9 (6.7)	3.4 (0.8)	P	0	382 (0.5)	na	4,544 (6.3)	4,162 (5.8)
Cambridge (UK)	24,818	59.3 (9.3)	3.7 (0.7)	C	0	637 (2.6)	0	10,199 (41.1)	9,562 (39.5)
Utrecht (NL)	16,869	57.7 (6.0)	4.4 (0.8)	P	0	225 (1.3)	859 (5.1)	4,723 (28.0)	3,639 (23.1)
Malmo (SW)	27,463	58.1 (7.6)	4.9 (0.5)	P	0	776 (2.8)	na	5,832 (21.2)	5,056 (18.9)
Doetinchem (NL)	7,139	44.0 (11.8)	5.0 (0.2)	C	1,101 (15.5)	68 (1.0)	80 (1.1)	2,584 (36.2)	1332 (22.6)
Denmark	55,753	56.7 (4.4)	5.3 (0.3)	P	0	1,610 (2.9)	317 (0.6)	11,594 (20.8)	9,667 (18.0)
Oxford (UK) *Health Conscious*	47,208	44.5 (14.5)	5.3 (0.5)	P	0	827 (1.8)	393 (0.8)	17,188 (36.4)	15,968 (34.7)
Oxford (UK) *General Health*	7,221	53.1 (7.9)	5.6 (0.6)	P	0	138 (1.9)	29 (0.4)	1,945 (26.9)	1,778 (25.2)
Norway	35,829	48.2 (4.3)	6.2 (0.5)	P	0	375 (1.0)	157 (0.4)	9,491 (26.5)	8,959 (25.4)
Amsterdam/Maastricht (NL)	14,920	42.3 (10.9)	6.2 (1.1)	P	0	163 (1.1)	533 (3.6)	5,483 (36.7)	4,787 (33.7)
Greece	25,997	53.2 (12.6)	7.7 (2.1)	T	0	590 (2.3)	na	1,832 (5.3)	792 (3.1)
Potsdam (GE)	26,891	50.5 (9.0)	8.5 (0.9)	P	4,282 (15.9)	827 (3.1)	na	8,528 (31.7)	3,419 (15.7)
Naples (IT)	4,947	50.3 (7.7)	8.7 (1.2)	P	4,271 (86.3)	1 (0.0)	0	4356 (88.1)	84 (12.4)
Florence (IT)	13,089	51.6 (7.7)	9.3 (1,1)	P	0	230 (1.8)	108 (0.8)	2,883 (22.0)	2,545 (20.0)
Umea (SW)	25,048	46.1 (10.3)	9.9 (0.3)	P	0	759 (3.0)	1012 (4.0)	11,501 (45.9)	9,730 (41.8)
Varese (IT)	11,797	51.6 (8.2)	11.1 (1.1)	P/T	0	287 (2.4)	209 (1.8)	1,749 (14.8)	1,253 (11.1)
Ragusa (IT)^d^	5,949	47.3 (7.6)	-	-	5,949 (100)	-	-	5,949 (100)	-
Turin (IT)^d^	9,749	50.2 (7.7)	-	-	9,749 (100)	-	-	9,749 (100)	-
**Total**	**497,969**	**51.5 (9.9)**	**5.3 (2.4)**		**25,355 (5.1)**	**8,226 (1.7)**	**3,697 (0.7)**	**122,154 (24.5)**	**84,876 (18.4)**

Abbreviation: yrs - years, SD – standard deviation, na - no information available, C - 2^nd^ assessment at study center; P - 2^nd^ assessment by postal survey; T - 2^nd^ assessment by telephone interview.

^a^ Multiple centers within a country were treated as a single study center, when length of follow-up and data collection methods did not differ and when coordination took place out of one center (Spain, France, Denmark). In Norway and Greece only one research center was situated.

^b^ Percentage corresponds to the overall response rates calculated according to the standard definitions used by the American Association for Public Opinion Research (AAPOR) [20].

^c^ For calculation of the proportion non-response, baseline participants who were not (yet) approached, died or emigrated before the second assessment were excluded (n = 37,278).

^d^ Ragusa and Turin did not contact any of their participants for a second assessment yet.

### Baseline characteristics of non-responders

Table 2 presents mutually adjusted associations between baseline characteristics and non-response. Non-responders were more likely to be male, to be young (≤40 years), to live alone (single, divorced/separated, widowed), to be less educated (primary or vocational secondary school), to be physically inactive (i.e. sedentary job and no recreational activity), to be current smokers, to be either a non or heavy alcohol user, to have a chronic disease at baseline but not cancer, to have developed cancer between baseline and second weight assessment and to have either a low (<18.5 kg/m^2^) or a high BMI (>25, especially ≥30 kg/m^2^). Based on the Wald statistic, the characteristics that strongly predicted non-response were recruitment strategy and duration of follow-up. A more active way of contacting participants, i.e. through direct telephone interview instead of a mailed questionnaire, resulted in a six-fold higher response. Non-response was more than three-fold if the follow-up assessment occurred after a long period, particularly more than 8 years.

**Table 2:** **Multivariate odds ratios of non-response to a second assessment of body weight in the EPIC-PANACEA study**^**a**^

	**Participants available for 2**^**nd **^**assessment (*n*** **= 460,691)**			
	**Number of participants *n***	**Non-responders (*n*** **= 84,876) n (%)**	**OR**^**b**^	**95% CI**
*Sex*				
Missing	0			
Male	130,030	26,190 (20.1)	1.0	
Female	330,661	58,686 (17.7)	0.92	0.90, 0.94
*Age*				
Missing	0			
≤40 years	50,789	14,911 (29.4)	1.0	
41-60 years	317,177	51,997 (16.4)	0.48	0.47, 0.49
>60 years	92,725	17,968 (19.4)	0.51	0.50, 0.53
*Marital status*				
Missing	106,239			
Married/living together	279,994	52,581 (18.8)	1.0	
Living alone^c^	74,458	19,077 (25.6)	1.4	1.37, 1.43
*Highest educational level*				
Missing	19,063			
College or university	110,246	18,893 (17.1)	1.0	
Other secondary school	100,618	15,447 (15.4)	0.93	0.90, 0.95
Vocational secondary school	104,299	23352 (22.4)	1.08	1.05, 1.10
Primary school or less	126,465	22,300 (17.6)	1.35	1.31, 1.38
*Cambridge physical activity index*				
Missing	65,002			
Active	73,596	13,859 (18.8)	1.0	
Moderately active	96,772	14,710 (15.2)	0.98	0.95, 1.01
Moderately inactive	137,438	20,802 (15.1)	0.99	0.96, 1.01
Inactive	87,883	14,593 (16.6)	1.15	1.12, 1.19
*Smoking status*				
Missing	9,722			
Never	227,854	37,297 (16.4)	1.0	
Former	122,690	23,209 (18.9)	1.02	1.00, 1.04
Current	100,425	22,452 (22.7)	1.33	1.30, 1.35
*Alcohol consumption*				
Missing	0			
Light alcohol users	302,995	59,630 (19.7)	1.0	
Non-users	61,316	10,467 (17.1)	1.18	1.15, 1.21
Moderate alcohol users	84,255	12,901 (15.3)	0.96	0.93, 0.98
Heavy alcohol users	12,125	1,878 (15.5)	1.12	1.06, 1.19
*Cancer at baseline*				
Missing	16,618			
No	423,646	76,910 (18.2)	1.0	
Yes	20,427	3,954 (19.4)	0.86	0.81, 0.91
*Chronic disease at baseline*^*d*^				
Missing	45,998			
Healthy	378,342	63,115 (16.7)	1.0	
Disease	36,351	7,027 (19.3)	1.33	1.27, 1.39
*Cancer before 2*^*nd*^*weight assessment*				
Missing	0			
No	448,198	81,752 (18.2)	1.0	
Yes	12,493	3,124 (25.0)	1.39	1.33, 1.45
*BMI*				
Missing	334			
18.5 - 25 kg/m^2^	236,019	43,404 (18.4)	1.0	
< 18.5 kg/m^2^	6,868	1,451 (21.1)	1.16	1.09, 1.23
25 - 29.9 kg/m^2^	157,093	29,072 (18.5)	1.08	1.06, 1.10
≥ 30 kg/m^2^	60,377	10,850 (18.0)	1.26	1.23, 1.29
*Recruitment strategy*				
Missing	0			
Postal survey	353,699	71,436 (20.2)	1.0	
Approached by elephone	76,924	2,546 (3.3)	0.16	0.15, 0.16
Requested to visit study center	30,068	10,894 (36.2)	2.7	2.58, 2.78
*Mean follow-up time*				
Missing	0			
0 - 4.0 years	160,750	15,867 (9.9)	1.0	
4.1 - 8.0 years	230,155	51,978 (22.6)	3.00	2.91, 3.06
8.1 - 12.0 years	69,786	17,031 (24.4)	3.26	3.16, 3.36

Abbreviations: OR - odds ratio; CI - confidence interval.

^a^ Participants who were not (yet) approached, died or (e)migrated during follow-up were not excluded in the present analyses.

^b^ A multivariate logistic regression model was used with response status (0 = responder; 1 = non-responder) as the dependent variable and all baseline characteristics, recruitment strategy and mean follow-up time as independent variables. All variables are mutually adjusted.

^c^ Single, divorced/separated, widowed.

^d^ Hypertension, stroke, myocardial infarction, diabetes mellitus.

When comparing characteristics of the baseline cohort to the characteristics of all persons who had a missing second anthropometric assessment (death, (e)migrated, not yet contacted and non-response) on average the same characteristics were related to missingness (data not shown). Participants for whom a second weight assessment was missing were more often male, young (<40 years), living alone (single, divorced/separated, widowed), less educated (primary or secondary school as highest attained educational level), former or current smokers, alcohol abstainers, chronically diseased at baseline, having developed cancer between baseline and second weight assessment and having either a low (<18.5 kg/m^2^) or a high BMI (>25, especially ≥30 kg/m^2^). A missing second body weight assessment was not related to heavy alcohol use or physical inactivity.

## Discussion

We investigated whether baseline demographic, socio-economic and health variables were different between responders and non-responders to a second assessment of body weight in a large European cohort. Our results suggest that non-response was non-random, but linked to specific characteristics of the participants at baseline. Both analyses, responders versus non-responders and responders versus all participants with missing second body weight assessment showed that non-responders were more often male, aged under 40 years, living alone, less educated, of poorer health, reported an unhealthy lifestyle and had either a low or a high BMI. Moreover, important predictors of a high response were a short follow-up time and an active way of follow-up using personal telephone interviews.

### Non-response

Overall response for the EPIC-PANACEA cohort was 81.6%, varying from 58.2% to 98.8% per center/country. One explanation for the generally high response is that all responders already were participants in the EPIC study assessing relations between lifestyle and chronic diseases. Usually this is a selective population with higher interest in medical and health issues and therefore higher motivation to participate in follow-up assessments. Follow-up time and recruitment strategy differed between the different study centers explaining part of the differences in response between the centers. A shorter time between first and second assessment was associated with a higher response, possibly because participants in centers with short follow-up duration still felt more involved in the study. Direct approach by telephone also yielded a higher response when compared to using a postal questionnaire. This implies that efforts of researchers to try and contact participants by telephone may enhance participation. However, requesting participants to visit a research center causes a burden to some persons, resulting in a lower response when compared to mailed or telephone administered questionnaires. However, we should be cautious with these conclusions because we did not study effects of different follow-up times and recruitments strategies within a center. So, center differences may also contribute to the observed differences in response between centers with different follow-up times and recruitments strategies. For example, in the Spain where participants where contacted by phone, blood donors were included and donors might be cooperative with regard to health related initiatives in general.

We identified several demographic and socioeconomic determinants that were independently related to non-response. The lower response among participants below age 40 years may relate to work obligations and family commitments and consequently less time to take part in research. This effect of age is in agreement with some studies [1-3,12], although others found non-response increasing with age [6,8,9]. Similar to what has been consistently reported in previous studies [1-4,6,9,13], we found lower response among participants living alone, i.e. single, divorced/separated or widowed. They depend on their intrinsic motivation to maintain participation in the study and might lack the encouragement of a partner. Furthermore, in agreement with others [3,13], who reported educational level as one of the most important predictors of non-response, low education was associated with lower response. Reporting a less healthy lifestyle at baseline, i.e., physical inactivity, being a smoker and both, either absence of alcohol or heavy use, was related to higher non-response. Similar results for alcohol use were reported by Thomas et al. [11], who suggested that three-quarters of alcohol abstainers were ex-drinkers, having given up drinking because of ill-health. Participants with a chronic disease at baseline were more likely to refuse participation in the follow-up survey, however, participants with cancer were more likely to respond. This might be due to the fact that the EPIC study was designed specifically to investigate causes of cancer and, therefore, (ex)cancer patients who already decided to participate at baseline were still willing to contribute to this study at a later time. In contrast, participants who developed cancer during follow-up were less likely to respond to an invitation for the second assessment.

Comparing responders with persons for whom a second body weight assessment was missing (i.e. death, (e)migrated, not yet contacted, non-responder) yielded rather similar results. This may imply that future studies that assess relations between baseline characteristics and weight changes during follow-up should consider the possibility of selective non-response. However selective non-response does not automatically imply selection bias and thus distorted effect estimates. If there is selection bias, statistical tools may help in adjusting, such as the method of 'inverse probability' [21,22]. Several other studies that investigated the magnitude of bias due to non-response showed that bias in these relationships was negligible [3,14].

Strengths of the present study are the large samples of participants from several European centres, the use of various methods of collecting the second assessment, different time intervals between the first and the second assessment and the use of standardized and validated baseline questionnaires across centres. Some limitations should be considered when interpreting our results. First, the determinants of attrition are based on baseline data, while some lifestyle factors might fluctuate or change over time. Second, in a cohort of half a million people misclassification of exact dates of vital status or migration may occur. Consequently, in some centers the non-response group may include persons already deceased or (e)migrated but not yet registered as such. Third, the selection of the study population in each EPIC centre was largely influenced by practical considerations. Therefore, the sample was not intended to be representative of each region and investigating cross-cultural differences in non-response was not possible. Finally, we studied the response to a second assessment of anthropometric measures. In many centers, at the same time other lifestyle or nutritional data were collected. The type of information as well as the amount may also affect response.

## Conclusions

In conclusion, in this large cohort study response to a second assessment, between 2–11 years after baseline, was reasonably high and varied between centers according to follow-up time and recruitment strategy. Non-response was more frequent in participants who were young, living alone, less educated, of poorer health, reported an unhealthy lifestyle and had either a low or high BMI. Cohort studies, especially those with long follow-up, may enhance cohort maintenance by paying extra attention to groups with above-mentioned characteristics who are most unlikely to respond and by an active recruitment strategy using telephone interviews.

## Competing interests

The authors declare that they have no competing interests.

## Authors' contributions

PHMP: principal investigator of the EPIC-PANACEA project and guarantor of the article; AMM, LEA and PHMP: conceived the current study; AMM, LEA, PHMP: responsible for the design of the study, analyses of data, interpretation of results; AMM, LEA drafting of the manuscript, taking into account the comments and suggestions of the coauthors; contributors from the collaborating centers (DR, ACV, AA, UK, AS, PO, NS, SR, TM, SR, SH, HB, MMB, MUJ, KO, NJW, CG, AT, JH, TJK, EAS, VH, JM, BH, EL, TB, FCC, MCBR, LR, MJS. MD, AB, JMH, AN, AT, DP, BP, TN, AM, RT, DA, HBBM, ER, PHMP): provided the original data, information on the respective populations, and advice on study design, analysis, and interpretation of the results; and all coauthors: had the opportunity to comment on the analysis and interpretation of the findings and approved the final version of the manuscript. All authors read and approved the final manuscript.

## Pre-publication history

The pre-publication history for this paper can be accessed here:

http://www.biomedcentral.com/1471-2288/12/148/prepub
